# Substrate-Dependent Optical Blue-Shift upon F Incorporation in Oxyfluoride SrCo(O,F)_3–*x*_ Films

**DOI:** 10.1021/acs.inorgchem.4c05324

**Published:** 2025-02-27

**Authors:** Tessa D. Tucker, Zongmin Yang, David Bugallo, Rajesh Dutta, Prajwal M. Laxmeesha, Gabriela A. Marrero-Hernández, Steven J. May

**Affiliations:** † Department of Materials Science and Engineering, Drexel University, Philadelphia, Pennsylvania 19104, United States; ‡ Department of Chemistry, University of Puerto Rico at Cayey, Cayey, Puerto Rico 00736, United States

## Abstract

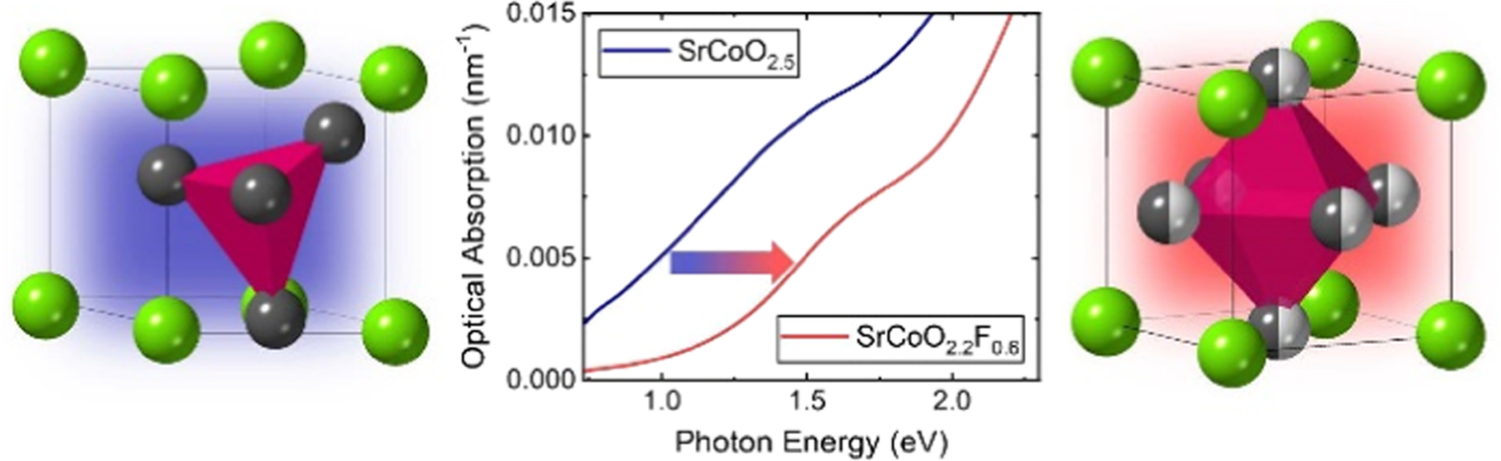

Heteroanionic oxides are attractive for their structural versatility and property tunability. In this work, we report on optoelectronic properties in epitaxial oxyfluoride SrCo(O,F)_3–*x*_ films synthesized on multiple (001)-oriented perovskite substrates. Topochemical fluorination was conducted on the as-grown SrCoO_2.5_ films at 200 °C using vapor from poly(vinylidene fluoride) (PVDF) as the fluoride source, producing films with a nominal SrCoO_2.2_F_0.6_ composition. Uniform fluoride insertion in each film was confirmed via depth-dependent elemental analysis, performed with X-ray photoelectron spectroscopy (XPS). Fluoride incorporation results in an expansion of the *c*-axis parameter, an increase in resistivity, and a blue-shift of the optical absorption edge by 0.18–0.5 eV. The magnitude of the band gap increase is strongly dependent on the in-plane lattice parameter of the substrate with larger blue-shifts observed in films grown on substrates with smaller lattice parameters, a trend that mirrors the larger resistivity enhancements present in the compressively strained oxyfluoride films. These results are attributed to the interplay between epitaxial strain and fluoride lattice site occupation, suggesting strain-dependent control of anionic arrangements is a promising route for engineering optoelectronic properties in heteroanionic films.

## Abstract 2

The substrate-dependence of fluorination reactions carried out on SrCoO_2.5_ films is presented. Upon fluorination, the SrCoO_2.2_F_0.6_ films exhibit a *c*-axis expansion, a resistivity increase, and a blue-shift of the optical absorption edge that is strongly strain dependent with compressive strain leading to a larger band gap expansion. These properties are attributed to preferential site occupation of F^−^, suggesting strain-dependent control of anionic arrangements is a promising route for controlling optoelectronic properties in heteroanionic films.

## Introduction

Anionic substitution is an emerging approach for tuning and expanding the functional properties of perovskites and other families of complex metal oxides.^1−3^ In these heteroanionic compounds, alloying with nitrogen or fluoride to synthesize oxynitrides or oxyfluorides can significantly change the covalency or ionicity of the metal-anion bond and the nominal oxidation state of the transition metal cation. For example, owing to the greater covalency of metal–N bonds compared to metal–O bonds, the valence band maxima of oxynitrides reside at higher energies compared to their pure oxide counterparts leading to a reduction of the band gap.^1,4,5^ This has positioned oxynitrides as promising materials for photocatalytic applications as their band gap can facilitate enhanced absorption within the solar spectrum.^6−8^ Conversely, in oxyfluorides the valence band states that are derived from fluoride anions reside at lower energies than the oxygen-derived states. Therefore, fluoride alloying may provide a means to increase band gaps of perovskite oxides, although direct comparisons between oxides and oxyfluorides in which the transition metal is in the same oxidation state are scarce.^9−12^

Much of the previous work establishing how anionic substitutions alter band gaps has been carried out either on bulk materials or through density functional theory studies of equilibrium structures. In contrast, much less is known regarding the electronic structure and band gaps of epitaxial films of heteroanionic materials, in which substrate-induced strain can impact anionic substitution. Heteroanionic films have been deposited directly from bulk targets of the parent compound in the case of oxynitrides^13−15^ or through postdeposition topochemical processes, such as reductive vapor-based fluorination reactions,^16−19^ ammonolysis for nitrogen incorporation,^20^ and voltage-driven fluoride insertion using ionic liquids.^21^ In films, epitaxial strain can result in anion-site ordering in both oxynitrides and oxyfluorides.^22−25^ Strain can also impact the kinetics of topochemical fluorination reactions where fluoride incorporation occurs via diffusion from the film surface. For example, the F^–^ concentration in SrMnO_3−δ_F_γ_ films was found to increase with increasing tensile strain in samples exposed to identical fluorination reaction conditions.^25^ However, it remains an open question how the confluence of both substrate-induced strain and F^–^ substitution impacts optical properties in oxyfluoride films.

In this work, we show that the optical absorption edge of oxide SrCoO_2.5_ and oxyfluoride SrCoO_2.2_F_0.6_ films exhibit opposing trends as a function of the substrate lattice parameters. While the band gap of SrCoO_2.5_ shows a slight red-shift with increasing compressive strain, upon fluorination the oxyfluoride films under compressive strain exhibit a much larger blue-shift compared to films under tensile strain. These optical trends are observed in films with similar F^–^ concentrations, directly confirming the role played by the substrate in altering the band gap of oxyfluoride films. These results illustrate how strain-dependent changes to electronic structures in oxyfluorides can differ markedly from their oxide counterparts.

## Experimental Methods

### Thin Film Growth

All epitaxial thin films discussed in this paper were synthesized via oxygen-assisted molecular beam epitaxy (MBE). Substrate preparation included sonication in acetone (15 min) followed by sonication in isopropanol (15 min) prior to mounting on a stub with silver paint. The 5 × 5 mm^2^ substrates were then swabbed with acetone and isopropanol before being introduced to the MBE load lock and transferred to the MBE main chamber. The SrCoO_2.5+δ_ (BM-SCO) films were deposited on (001)-oriented (La,Sr)(Al,Ta)O_3_ (LSAT), GdScO_3_ (GSO), SrTiO_3_ (STO), and LaAlO_3_ (LAO) single crystal substrates (MTI Corporation). The Co source was heated to ∼1350 °C in a W crucible and Sr source heated to ∼405 °C in a pyrolytic boron nitride (PBN) crucible for codeposition. Deposition rates were determined using a quartz crystal microbalance (QCM), calibrated through Rutherford backscattering spectrometry (RBS) of reference films. We estimate an error of ∼2% for the Sr:Co ratio of the as-grown films based on uncertainty of the RBS experiment, analysis, and flux measurement within the MBE. Oxygen was introduced to the chamber while substrates were being heated to a growth temperature of approximately 650 °C for thin film synthesis. The O_2_ environment was maintained at a pressure of ∼5 × 10^–6^ Torr throughout growth. The deposition rate was approximately one unit cell per minute, with each layer deposition consisting of a 10 s pause after Sr and Co deposition. *In situ* reflection high-energy electron diffraction (RHEED) was used to monitor in-plane crystallinity. Film thicknesses ranged from 29 to 33 nm. We note that RBS evaluation of BM-SCO films grown around this time contained trace amounts of W (approximately 0.01:1 W:Co) that we attribute to slight alloying of Co with the W crucible. However, this does not appear to impact physical properties as our structural, optical, and electronic data of BM-SCO films is consistent with the literature as described in the results section.

### Topochemical Fluorination

**Figure 1:**
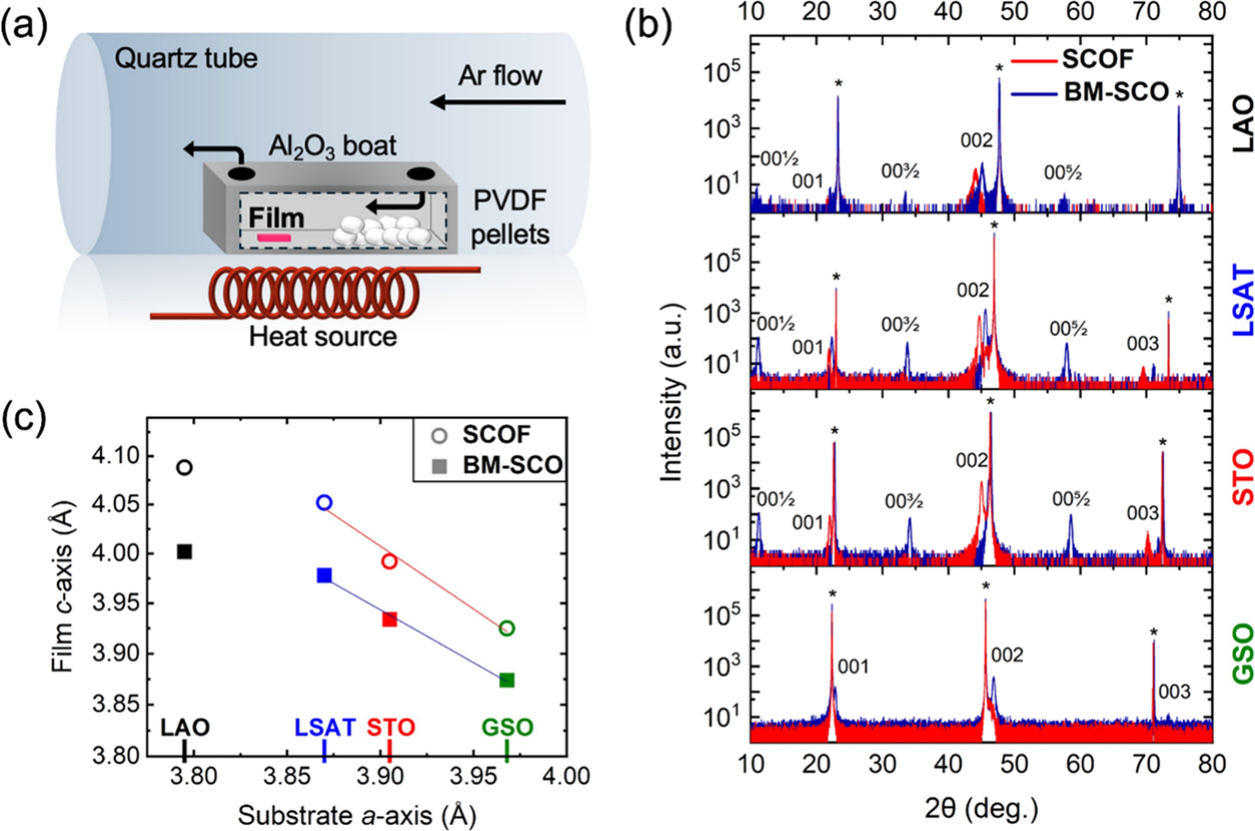
(a) Fluorination schematic. (b) XRD scans measured before and after fluorination on each epitaxial film where the asterisk indicates the respective substrate diffraction peak. (c) The pseudocubic *c*-axis lattice parameter as a function of substrate *a*-axis.

Fluorination was performed in a quartz tube furnace as shown schematically in Figure 1a. Films were placed downstream from poly(vinylidene fluoride) (PVDF) pellets in an alumina boat wrapped in aluminum foil. Two holes were punctured in the aluminum foil above the film and PVDF to allow gas exchange. The boat was then placed in the center of the tube furnace. The gas lines and quartz tube were purged with Ar for 5 min at 1 lpm, then the flow rate was reduced to 0.25 lpm which was maintained throughout the process. At a rate of 15 °C/min, the furnace was heated to 200 °C and remained at temperature for 1.5 h. The Ar flow was maintained throughout the reaction and afterward until the sample had cooled to 50 °C.

### Structural and Compositional Characterization

X-ray diffraction (XRD), reciprocal space maps, and X-ray reflectivity (XRR) was performed using a Rigaku Smartlab X-ray diffractometer. Data was taken in parallel beam mode using a Ge (220) double-bounce high-resolution monochromator and 5 mm slit. XRR data was simulated and fit by GenX.^26^ Thin film *c*-axis parameters were calculated using Bragg’s law based on the 002 diffraction peak position. X-ray photoelectron spectroscopy (XPS) was performed with a PHI VersaProbe 5000 and Ar^+^ sputtering for depth profiling. One survey scan and individual elemental spectra were collected presputter. Sputter time was set to 15 min with 30 s intervals and 30 s delayed acquisition time. Elemental analysis included C, Sr, Co, O, F, and the elements in each substrate. Carbon spectra was used to calibrate binding energies. Pass energy was set to 46 eV, with energy steps of 0.1 eV and 100 ms per step. Elemental spectra were repeated twice. XPS spectra and depth profiles were analyzed using CasaXPS software.

### Electronic Characterization

Resistance as a function of temperature was measured using a Quantum Design Physical Property Measurement System (PPMS) and an external Keithley precision current source and nanovoltmeter. Wires were soldered to sample puck channels and contacts were made to the film with silver paste in the Van der Pauw geometry. Resistance was converted to resistivity (ρ) for each film using  where *t* is the thickness of the film.

### Optical Characterization

The refractive index and extinction coefficient, *n* and *k*, of the thin films were extracted from optical data measured using a J. A. Woollam variable-angle spectroscopic ellipsometer. Measurements were taken from 65 to 75° and over an energy range of 0.74–5.05 eV. Data was modeled and fit using CompleteEase software at a resolution of 0.1 eV. Bare substrates and thin films were measured and fit using Cauchy and B-spline dispersion models, respectively. The optical absorption coefficient (α) was calculated using α = 4π*k*λ^–1^, where λ is the photon wavelength.

## Results

A schematic of the fluorination process is shown in Figure 1a. This partially reductive process is limited by diffusion of fluoride ions from the surface to the bulk of the brownmillerite SrCoO_2.5+δ_ (BM-SCO) films, producing SrCo(O,F)_3–*x*_ (SCOF) films. To achieve maximum fluoride content through diffusion and avoid film degradation, a balance is required between the time and temperature at which fluoride ion incorporation is performed. Fluorination reactions used in this study were performed at 200 °C for 1.5 h. Reactions run for longer times did not result in incorporation of additional fluoride, suggesting that this process is then no longer kinetically limited under these conditions, but is instead thermodynamically limited. In the as-grown films, Co has a nominal valence state of 3+ assuming a stoichiometry of brownmillerite SrCoO_2.5_.^27^ The vapor-based topochemical fluorination reaction is known to concurrently remove oxygen from the host material while fluoride is incorporated.^28^ This results in oxyfluorides that are either slightly reduced or do not exhibit a measurable change in oxidation state of the transition metal cation.^16,29^

The structural effects of substate-induced strain and fluoride incorporation were investigated using X-ray diffraction measurements carried out prior to and after topochemical fluorination. Figure 1b shows XRD results before and after fluorination, revealing differences in the films’ crystal structure between varying substrates and structural transformations due to fluorination. All as-grown films, except for the film synthesized on GSO, display “half-order” XRD peaks consistent with the brownmillerite structure.^30−32^ The brownmillerite structure is distinguished by the presence of ordered oxygen vacancies alternating along adjacent (001) planes, relative to a pseudocubic perovskite structure, along with antiferroic displacements of the *A*-site cations along the [001] direction. These two structural features combine to double the perovskite unit cell along the [001] direction, giving raise to “half-order” reflections such as 0 0 1/2 when indexed relative to a pseudocubic perovskite cell. Therefore, these reflections are confirmation of the brownmillerite structure and indicate the direction of vacancy ordering. A possible explanation for the absent half-order peaks in BM-SCO/GSO could be due to lower overall intensity resulting in undetectable half-peaks or there is ample oxygen vacancy disorder within the film.^30^ The *c*-axis lattice parameter was calculated using the pseudocubic 002 Bragg peak. We first discuss the effects of film/substrate lattice mismatch on the *c*-axis of the as-grown BM-SCO films under compressive (negative lattice mismatch) and tensile (positive lattice mismatch) strain. The lattice mismatch (*f*) between films’ in-plane lattice parameters and those of the substrates is calculated using *f* (%) = 100 × (*a*_s_ – *a*_f_)/*a*_s_, where *a*_s_ and *a*_f_ are the substrate and film in-plane lattice parameters, respectively. Lattice parameters for the substrates are LAO *a* = 3.795 Å, LSAT *a* = 3.870 Å, STO *a* = 3.905 Å, and pseudocubic GSO *a* = 3.968 Å; the pseudocubic bulk *a*-axis parameter for BM-SCO is 3.904 Å and the lattice parameter along the vacancy ordering direction (*c*-axis in this case) is 3.945 Å.^33,34^ This results in a 1.6% lattice mismatch within the film synthesized on GSO, and −2.8 and −0.9% mismatch values for the BM-SCO films on LAO and LSAT, respectively. The in-plane constraints on the lattice parameters of the film result in linear changes to the measured *c*-axis parameter as a function of *f* for the films on LSAT, STO, and GSO as shown in Figure 1c. The film with the largest negative lattice mismatch (BM-SCO/LAO), exhibits a *c*-axis parameter of 4.002 Å while the film on GSO, under the largest tensile strain, has a *c*-axis parameter of 3.874 Å. The film on LAO exhibits a *c*-axis that is less than the linear trend from the films on the other three substrates. This implies some degree of strain relaxation in BM-SCO/LAO, although the data suggests that this film is still strained given the expansion of the *c*-axis compared to bulk BM-SCO. Reciprocal space maps measured from the SCO/LAO and SCO/LSAT samples (presented in Supporting Information) support our interpretation that the as-grown films on LSAT, STO, and GSO are strained, while those on LAO are partially relaxed. While the SCO/LSAT film has an *a*-axis parameter identical to the LSAT substrate, the as-grown SCO/LAO film is partially relaxed with an in-plane lattice parameter of 3.879 Å. Previous work has demonstrated that this low temperature fluorination reaction does not lead to strain relaxation in perovskite films, thus we assert that the SCOF on LSAT, STO and GSO are strained.^19,25,29^ Given that the as-grown SCO/LAO film is partially relaxed, the fluorinated SCOF/LAO film must also be partially relaxed.

Following fluorination, the *c*-axis parameter expands in all films due to longer metal-F bond lengths compared to metal-O bonds.^25,29^ This result is observed through X-ray diffraction, showing a shift of the 00*L* diffraction peaks to smaller angles as seen in Figure 1b, consistent with other oxyfluoride studies.^16,18,25,29,35^ Additionally, the half-order diffraction peaks associated with the brownmillerite structure are no longer present after fluorination reflecting a transition from BM-SCO to pseudocubic perovskite SCOF. The full trend of *c*-axis as a function of substrate lattice parameter for as-grown oxides and oxyfluorides is shown in Figure 1c. Structurally, the *c*-axis elongation due to fluoride incorporation is largest (0.087 Å) for the film on LAO. In contrast, the film on GSO exhibits the smallest difference in *c*-axis elongation of 0.051 Å. The strain-dependent *c*-axis elongation suggests fluoride ions preferentially reside on the apical site of the octahedra for compressively strained films and the equatorial site for tensilely strained films.^25^ The larger length of Co–F bonds versus Co–O bonds better facilitates a *c*-axis elongation (compressive strain) when F^–^ occupies the apical site or conversely an in-plane elongation (tensile strain) when F^–^ resides in equatorial sites. Hence, the decrease in *c*-axis elongation with respect to increasing tensile strain in SCOF films is consistent with preferential occupation of F^–^ within the lattice.

**Figure 2:**
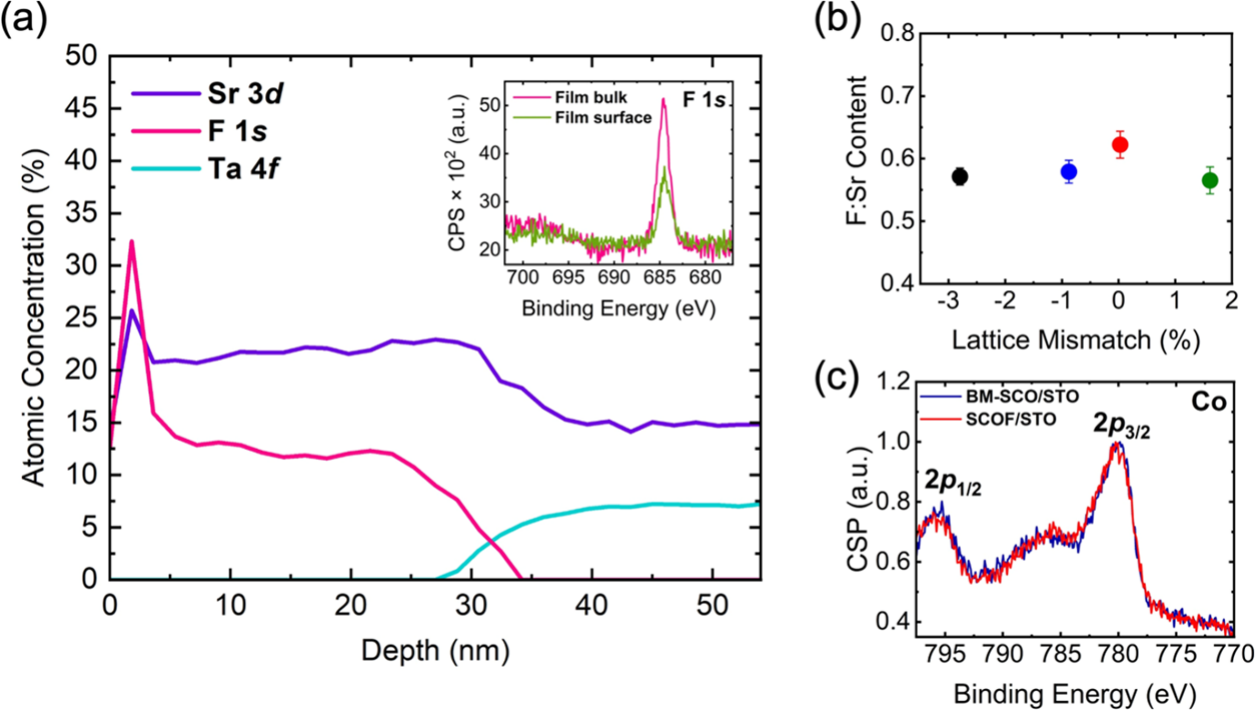
(a) SCOF/LSAT XPS depth profile of Sr, F, and Ta; inset shows F spectra at the surface and middle of film on LSAT. (b) F:Sr content (average of Sr and Co at 7 and 14 nm below the surface for each film) with respect to lattice mismatch. Error bars indicate the standard deviation obtained from measuring the F:Sr at multiple sputter depths. (c) Normalized Co 2p spectra from ∼7 nm below the surface in BM-SCO and SCOF on STO.

XPS was performed to determine the relative F^–^ concentration and uniformity within the films, as well as to determine the change in Co oxidation state in brownmillerites and perovskites. XPS depth profiling presented in Figure 2a shows the presence of fluoride throughout the SCOF film (30 nm) for the sample on LSAT. XPS depth profiles of all oxyfluoride films are presented in the Supporting Information. The F 1s peak is shown inset in Figure 2a for SCOF/LSAT at the surface of the film (0 nm) and within the bulk of the film (∼15 nm). The F 1s peak position is consistent with fluoride bonded to a transition metal. In contrast, fluoride bonded to organics such as in PVDF results in a peak near 688.8 eV, which is not present in these samples.^36^ The largest fluoride concentration appears ∼2 nm into the film, where sputtering most likely eliminated surface contamination from the film surface. Near-surface fluoride accumulation is a result of the diffusive nature of the fluorination process and was observed in previous works.^16,19^ As the etching continues past 2 nm, fluoride remains present until substrate elements are detected. As shown in Figure 2b, all films exhibit comparable F^–^ content regardless of substrate-induced strain. The fluoride content was determined through analysis of the XPS peak intensities. Assuming a 1:1 Sr:Co composition, the F concentration was calculated from the Sr:F intensity ratio. The fluoride content of each film was then averaged based on spectra obtained throughout the depth profile. The Co 2p spectra is shown in Figure 2c where no significant change is observed following the transition from BM-upon topochemical synthesis, indicating that the nominal Co oxidation state is unchanged after fluorination. The photoemission spectra are consistent with previously published data on SrCoO_2.5_,^37^ suggesting that the Co cations are nominally 3+. However, we acknowledge the complexity of interpreting Co photoemission spectra^38^ and the possibility of sputtering-induced reduction. Therefore, we cannot rule out the possibility of a mixed 3+/2+ state. Based on the majority presence of Co^3+^, we estimate the overall composition of the oxyfluorides to be SrCoO_2.2_F_0.6_.

**Figure 3:**
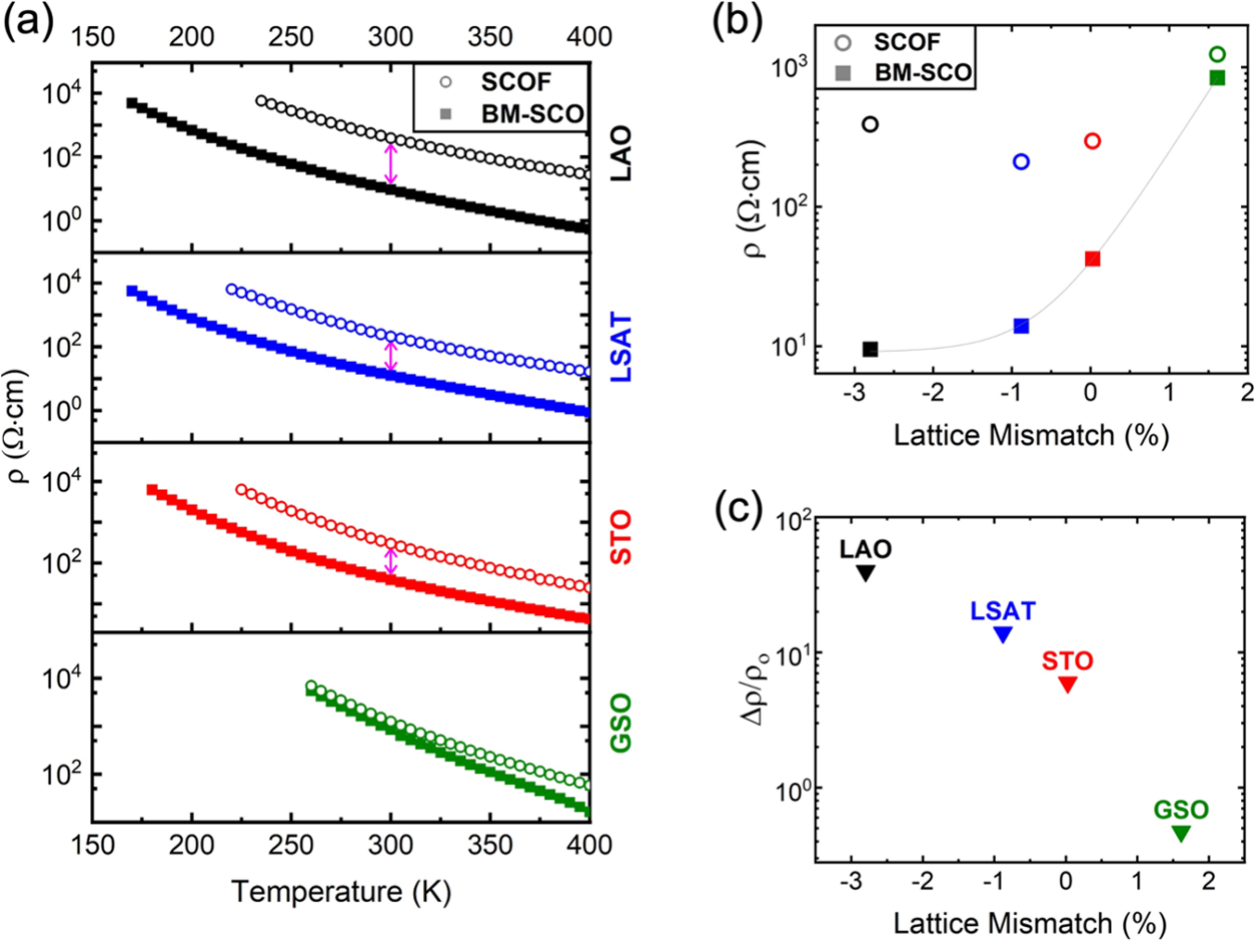
(a) Resistivity as a function of temperature for each film before and after fluorination. (b) Resistivity as a function of lattice mismatch in BM-SCO and SCOF at 300 K. (c) Change in resistivity upon fluorination divided by BM-SCO resistivity at 300 K for films as a function of lattice mismatch.

Electronic resistivity measurements as a function of temperature were carried out on films before and after fluorination. Figure 3a shows the resistivity of each film on the different substrates. The resistivity decreases as the in-plane lattice parameter of the substrate decreases for the as-grown BM-SCO films. The general insulating behavior present in all BM-SCO films is in good agreement with previous studies.^27,39^ At room temperature (300 K), BM-SCO/GSO shows the largest resistivity (ρ = 840 Ω·cm) followed by BM-SCO/STO (ρ = 39 Ω·cm), BM-SCO/LSAT (ρ = 13 Ω·cm) and BM-SCO/LAO, which exhibits the smallest resistivity, ρ = 9.5 Ω·cm. These results are consistent with previous reports of brownmillerites which showed that increased tensile strain results in a larger band gap and increased resistivity.^32,40^

As shown in Figure 3b, fluoride incorporation in BM-SCO films leads to an increase in resistivity. When comparing the change in resistivity divided by the initial resistivity (Figure 3c), a strong substrate-dependence is observed. As compressive strain decreases and tensile strain increases, the magnitude of the change in resistivity upon fluorination becomes smaller. Since there is a lack of substrate-dependence with respect to F^–^ content, the resistivity results do not arise from a simple correlation with F^–^ concentration. Instead, the resistivity trend is most likely a result of the strain-dependent preferential occupancy of fluoride anions. Fluoride located at the apical site in the SCOF films appears to yield a higher resistivity. Similar behavior was also observed in strained oxyfluoride SrMn(O,F)_3–*x*_ films, where the resistivity changes upon fluorination were much larger in films under compressive strain compared to films under tensile strain and where X-ray linear dichroism confirmed the strain-dependent occupancy of F^–^ in the apical and equatorial sites.^25^ We attempted to extract an activation energy from the resistivity data, which yielded values between 15 and 40 meV for all the oxide and oxyfluoride samples. However, a simple activated model only fits the data from all samples over a relatively limited temperature range of ∼260–315 K, and thus we refrain from drawing physical conclusions based on these values.

**Figure 4:**
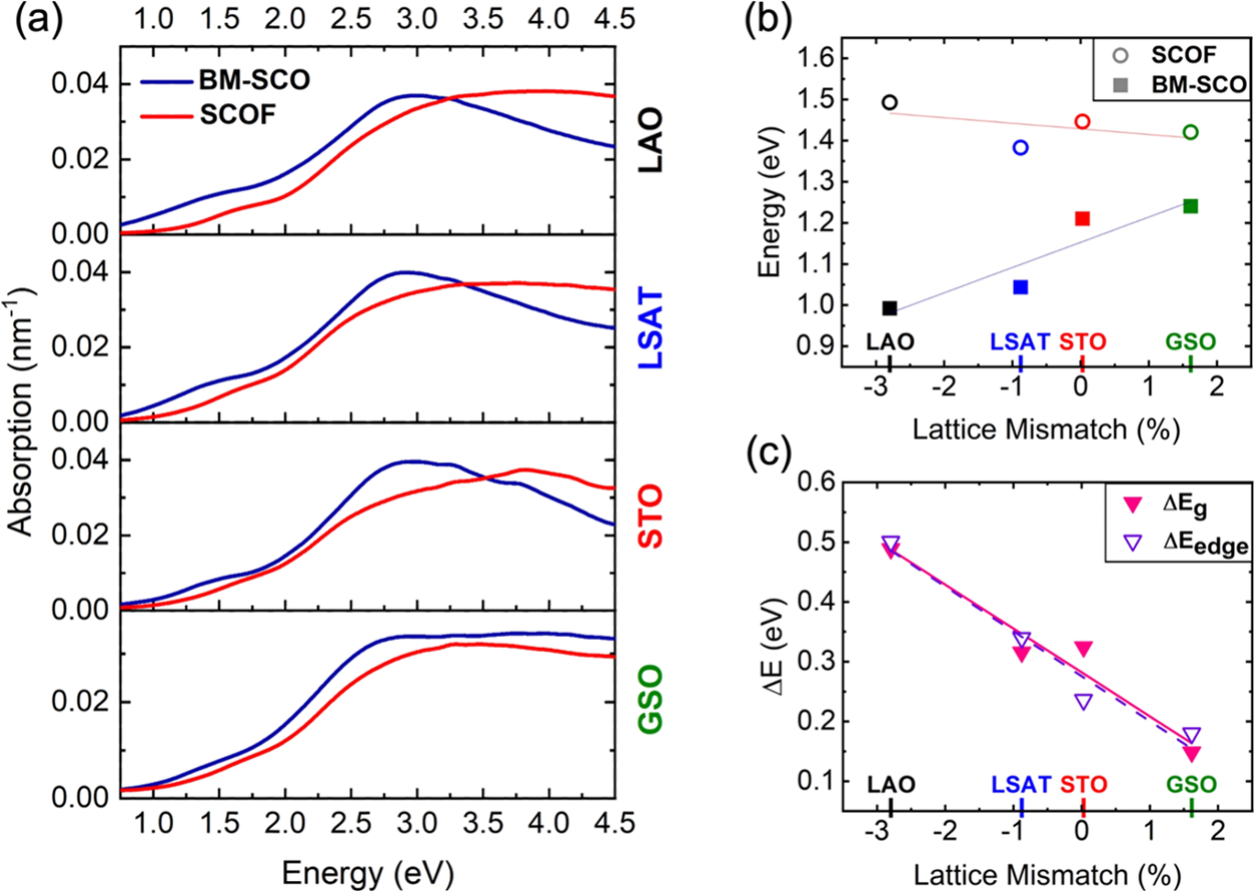
(a) Optical absorption spectra before and after fluoride incorporation. (b) Absorption onset energy where α = 0.005 nm^–1^ for BM-SCO and SCOF films. (c) Change in energy of BM-SCO and SCOF films calculated using absorption onset energy method (open symbols) and Tauc plot method (closed symbols).

The choice of substrate also plays a striking role in the changes to the optical band gap brought on by fluorination. In as-grown BM-SCO films, the optical band gap increases as the in-plane lattice parameter of the substrate increases (Figure 4). Upon fluorination, the optical absorption edge of SCOF films shows a blue-shift—a shift of the absorption edge toward higher energies. The magnitude of the blue-shift is strongly dependent on the strain state of the SCOF film as can be seen in Figure 4a. The nature of the band gap—indirect or direct—in SrCoO_2.5_ is not well established and therefore extraction of a band gap from the absorption spectra carries significant uncertainty as it requires one to assume a direct or indirect Tauc model. This analysis is presented in Supporting Information for as-grown and fluorinated films assuming an indirect Tauc model.^27,41^ Because the Tauc method can be ambiguous,^42^ an additional method of analysis was employed using the energy at which the absorption coefficient (α) corresponds to a specific value. Figure 4b shows the energy at which the absorption coefficient of BM-SCO and SCOF films is equal to 0.005 nm^–1^. This absorption value lies at the onset of the first absorption peak, in the same energy region where a line is fit to the slope of a Tauc plot. The blue-shift magnitude is then calculated by the energy difference between the fluorinated absorption edge and the as-grown absorption edge at α = 0.005 nm^–1^. As depicted in Figure 4c, both methods are in agreement regarding the substrate-dependent trend. For the remainder of the optical analysis, we will discuss the energy of absorption onset as it is less ambiguous than the Tauc plot method.

Lattice mismatch and fluoride incorporation simultaneously contribute to changes in SCOF films’ optical properties. An optical blue-shift of 0.18–0.50 eV is observed across SCOF films upon fluoride incorporation, with the largest shift (0.50 eV) occurring on LAO and the smallest shift (0.18 eV) occurring on GSO. The SCOF films on LSAT and STO showed a shift of 0.34 and 0.24 eV, respectively. These results point to a strain-dependence on the change in the optical absorption edge blue-shift upon fluorination. Previous reports have established that oxidation of SrCoO_2.5_ to SrCoO_3_ results in a red-shift and a closing of the gap with increasing oxidation of Co.^27,43^ In contrast, fluorination in which the transition metal is not oxidized has been shown to induce an absorption shift to higher energies in other oxyfluorides, such as SrFe O_2_F, where an optical blue-shift of less than 0.2 eV is observed upon fluorination of brownmillerite SrFeO_2.5_.^9,44^ Thus, our results are consistent with, but larger in magnitude than, previously observed fluorination-induced changes to band gaps in oxyfluoride perovskite films.

When comparing the optical absorption results to the resistivity measurements, the same general trend is present. As tensile (compressive) strain increases, the magnitude of the absorption edge shift and resistivity increase upon fluorination become suppressed (enhanced). These trends suggest that the site occupancy—F preferring apical (equatorial) occupancy under compressive (tensile) strain—likely plays a central role in determining optical absorption in oxyfluorides as the large differences in band gap observed in these films occurs across samples with the same F concentration. Future studies using X-ray linear dichroism would be beneficial for confirming this scenario. In addition to preferential occupancy, anion vacancies and their location within the lattice of these oxyfluorides could influence the suppression or enhancement of the properties. For instance, it is known that tensile strain decreases the formation energy for oxygen vacancies in perovskite oxide films.^45−47^ Similar effects could be at play in oxyfluorides, which warrants future investigation.

## Conclusions

We have investigated the substrate-dependence of optical, electronic, and structural properties in MBE-synthesized brownmillerite SrCoO_2.5+δ_ and perovskite SrCo(O,F)_3–*x*_ films. With increasing substrate lattice parameter, BM-SCO films show an increase in optical absorption edge energy and resistivity. Through topochemical synthesis, films were converted from SrCoO_2.5_ to SrCoO_2.2_F_0.6_. Upon fluoride incorporation in approximately 1/5 of the anion sites, the oxyfluorides exhibit a *c*-axis elongation, an optical blue-shift of the band gap, and increased resistivity. These fluoride-induced changes are strongly dependent on the substrate, with compressive strain leading to much larger increases in the band gap and resistivity. However, the lattice mismatch does not significantly alter the amount of F^–^ incorporated within the films. This leads us to conclude that a strain dependence of the fluoride site occupancy within the lattice is the origin of the property differences. This work highlights how strain can influence the properties of heteroanionic films in ways not accessible in pure oxide films, demonstrating the additional tuning parameters for property control in epitaxial oxyfluorides.

## Data Availability

The data in this paper can be accessed at https://doi.org/10.13011/m3-8148-8x36.

## Supporting Information Available

Full photoemission depth profiles of all SCOF samples, refractive index (*n*) and extinction coefficient (*k*) determined from ellipsometry for BM-SCO and SCOF samples, reciprocal space maps, and more information on optical absorption analysis (PDF)
