# Supplementary material for: Substrate-Dependent Optical Blue-Shift upon F Incorporation in Oxyfluoride SrCo(O,F)3–x Films
Source: Inorg Chem. 2025 Feb 27;64(9):4483–90. doi: 10.1021/acs.inorgchem.4c05324 (PMC11898045; doi:10.1021/acs.inorgchem.4c05324)
Supplement: Supplementary file 1 — ic4c05324_si_001.pdf [file ic4c05324_si_001.pdf]

Supporting Information for:

Substrate-Dependent Optical Blue-Shift Upon F Incorporation in Oxyfluoride $SrCo(O,F)_{3-x}$ Films

Tessa D. Tucker,[1] Zongmin Yang,[1] David Bugallo,[1] Rajesh Dutta,[1] Prajwal M. Laxmeesha,[1] Gabriela A. Marrero-Hernández,[1,2] Steven J. May[1,*]

[1] Department of Materials Science and Engineering, Drexel University, Philadelphia, PA 19104

[2] Department of Chemistry, University of Puerto Rico at Cayey

[*] smay@drexel.edu

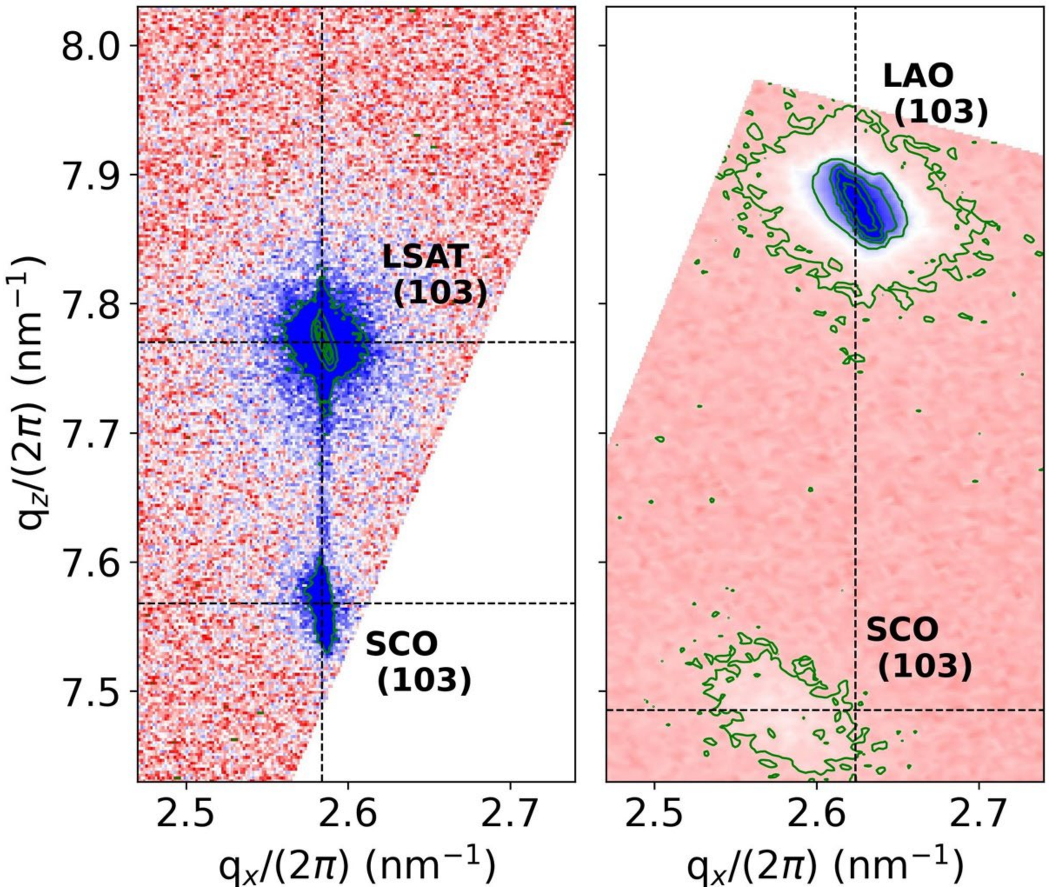

Figure S1. Reciprocal space maps from the as-grown SCO/LSAT and SCO/LAO films showing that the SCO/LSAT film is strained but the SCO/LAO film is partially relaxed.

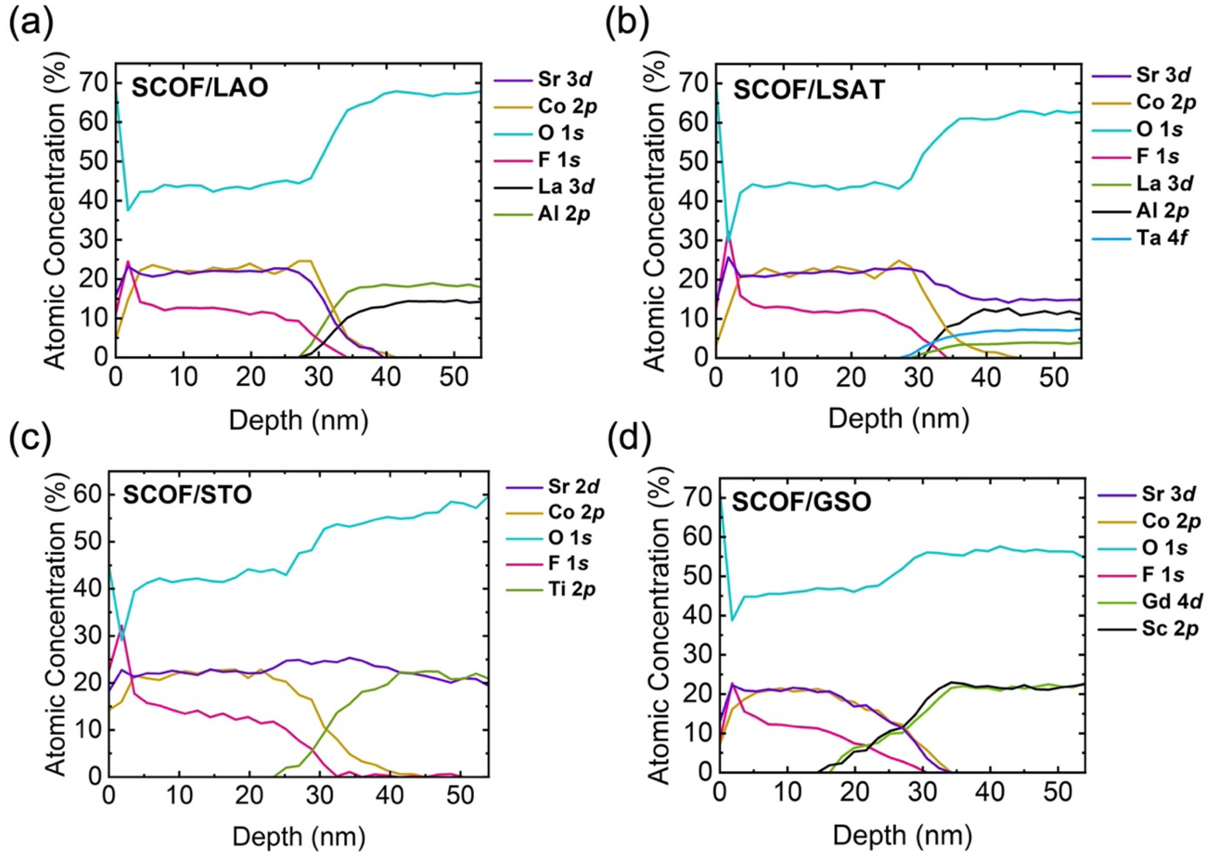

Figure S2. XPS depth profiles of SCOF films on (a) LAO, (b) LSAT, (c) STO, and (d) GSO.

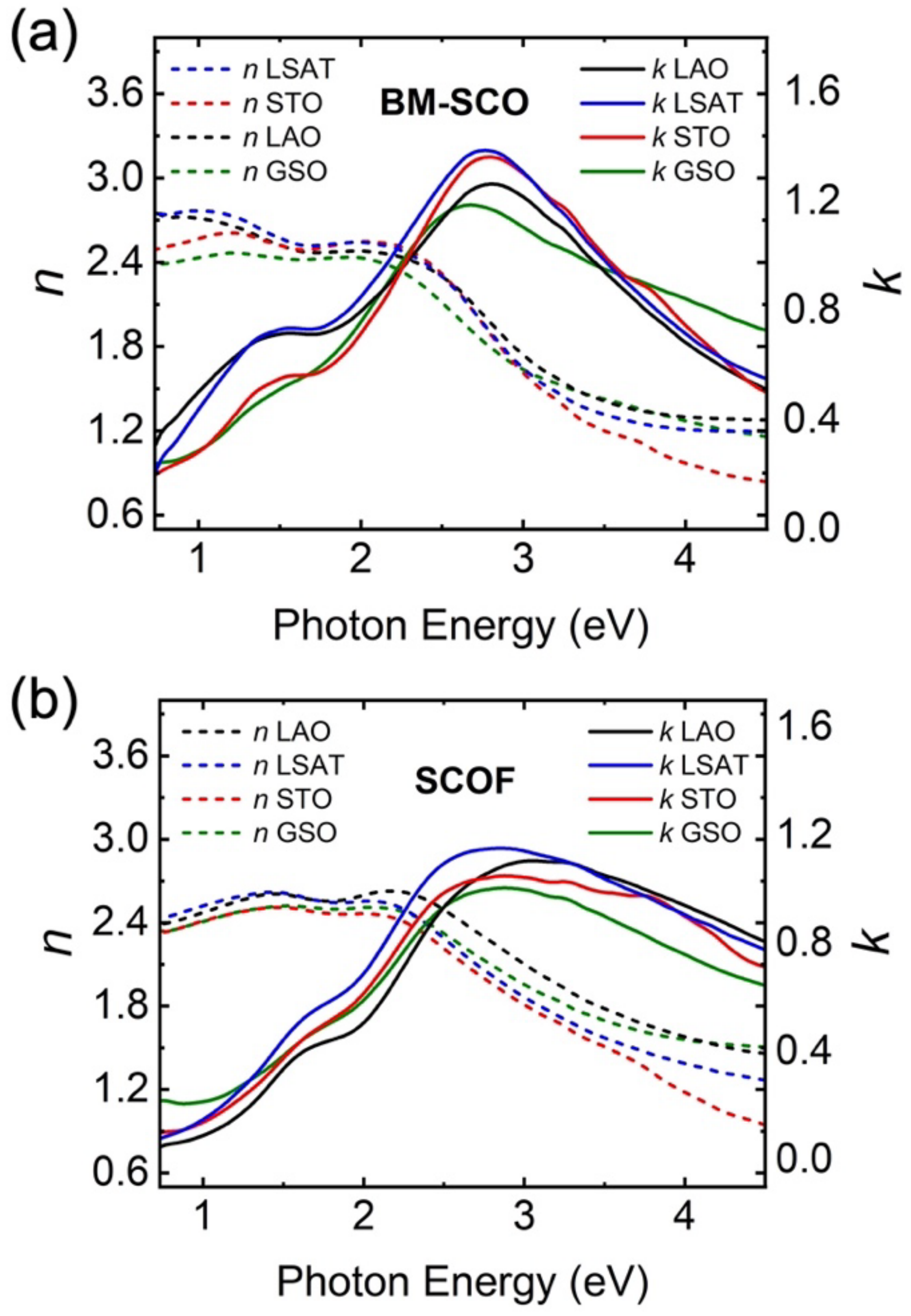

Figure S3. Refractive index, *n*, and extinction coefficient, *k*, of (a) BM-SCO and (b) SCOF films.

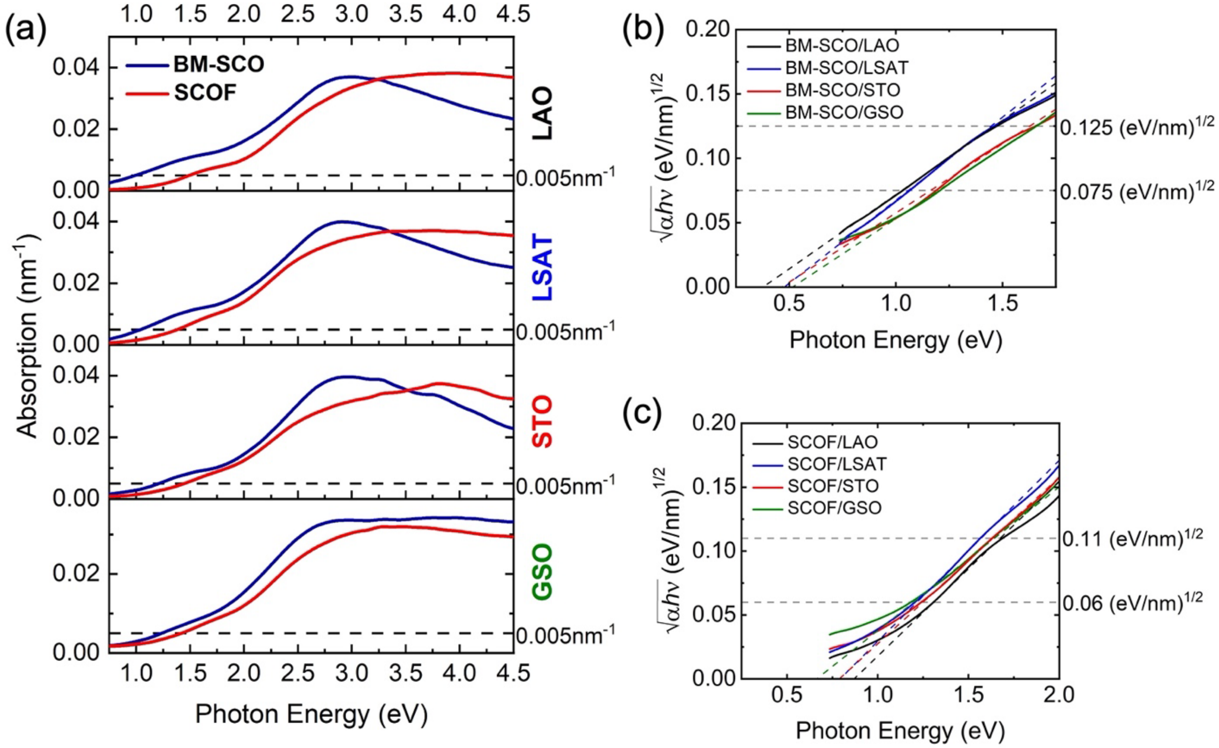

Figure S4. (a) Optical absorption spectra for BM-SCO and SCOF films on different substrates with the absorption at 0.005 nm^{-1} highlighted by the dashed horizontal line. (b) Tauc plot for BM-SCO films where linear fits reside within range of 0.075-0.125 $(eV/nm)^{1/2}$. (c) Tauc plot for SCOF films where linear fits reside within range of 0.06-0.11 $(eV/nm)^{1/2}$. Tauc analysis assumes an indirect band gap.
